# Grazoprevir and Elbasvir in Patients with Genotype 1 Hepatitis C Virus Infection: A Comprehensive Efficacy and Safety Analysis

**DOI:** 10.1155/2017/8186275

**Published:** 2017-01-09

**Authors:** Yinan Yao, Ming Yue, Jie Wang, Hongbo Chen, Mei Liu, Feng Zang, Jun Li, Yun Zhang, Peng Huang, Rongbin Yu

**Affiliations:** ^1^Department of Epidemiology and Biostatistics, School of Public Health, Nanjing Medical University, Nanjing 211166, China; ^2^Department of Infectious Diseases, The First Affiliated Hospital of Nanjing Medical University, Nanjing 210029, China; ^3^Department of Basic and Community Nursing, School of Nursing, Nanjing Medical University, Nanjing 211166, China; ^4^Department of Infectious Diseases, The Jurong People's Hospital, Jurong 212400, China; ^5^Department of Epidemiology, Medical Institute of Nanjing Army, Nanjing 210002, China

## Abstract

*Background.* It is urgent for patients with hepatitis C virus (HCV) infection to find a safe, effective, and interferon-free regimen to optimize therapy. A comprehensive analysis was performed to evaluate the efficacy and safety of the grazoprevir combined with elbasvir, with or without ribavirin (RBV), in 777 treatment-naive and treatment-experienced patients with HCV genotype 1 infection from 3 randomized controlled trials (RCTs).* Method.* We collected data from the following trials: C-WORTHY (NCT01717326), C-SALVAGE (NCT02105454), and C-EDGE (NCT02105467). All patients received grazoprevir plus elbasvir with or without RBV for 12 or 18 weeks. The sustained virological response (SVR) 12 weeks after end of treatment was calculated for overall and subgroups.* Results.* 568 (73%) patients were treatment-naive. Overall, 95% (95% CI: 93–96) patients achieved SVR12, 95% (95% CI: 92–96) for treatment-naive and 96% (95% CI: 92–98) for previously treated patients, respectively. Treatment duration and treatment regimen did not have great difference in SVR12 rates. The most common AEs were fatigue (18%–29%), headache (20%), nausea (8%–14%), and asthenia (4%–12%). One patient (<1%) receiving grazoprevir plus elbasvir alone and one (<1%) receiving grazoprevir plus elbasvir plus RBV had treatment-related serious AEs.* Conclusions.* The result shows that 12-week grazoprevir plus elbasvir therapy is safe and effective for treatment-naive patients with HCV genotype 1.

## 1. Introduction

Hepatitis C virus (HCV) infection is one of the major global health problems affecting all countries. According to recent estimates, 80–185 million people are infected with HCV worldwide [1, 2]. Chronic HCV infection gives rise to cirrhosis, hepatocellular carcinoma, hepatic decompensation, and liver transplantation [3]. Effective therapy reduces complications and mortality related to HCV infection [4]. These facts illustrate the growing medical need of effective regimens for patients with chronic HCV infection.

The first-line therapies approved for chronic HCV genotype 1 infection patients are sofosbuvir plus peginterferon plus ribavirin and simeprevir plus peginterferon plus ribavirin. The SVR rates were 92% in treatment-naive patients without cirrhosis (Metavir fibrosis stage F0–F2) and 80% in those with cirrhosis (Metavir fibrosis stage F4) treated with sofosbuvir plus peginterferon plus ribavirin [5]. In patients treated with simeprevir, peginterferon, and ribavirin, SVR rate in treatment-naive patients infected with HCV genotype 1 was 83–85% without cirrhosis but 58–65% with cirrhosis and 53% in treatment-experienced patients who had null responses to previous treatment [6–8]. The only available oral regimen for patients with HCV genotype 1 is 24 weeks of sofosbuvir plus ribavirin [9, 10]. The SVR rate for this regimen was only 68% overall in treatment-naive patients infected with HCV genotype 1 and without cirrhosis. However, SVR reduced to 50% in patients with advanced fibrosis [9]. In conclusion, regimens with peginterferon plus first-line protease inhibitors plus ribavirin are less effective and worse tolerated in patients with cirrhosis [11]. Therefore, an interferon-free, all-oral, short-duration, and effective HCV therapy is highly needed for all kinds of patients.

We performed this post hoc analysis in order to better determine the safety and efficacy of grazoprevir (an HCV NS3/4A protease inhibitor) plus elbasvir (an HCV NS5A inhibitor) in patients with HCV genotype 1 infection as well as provide the evidence for choosing the optimal treatment regimen.

## 2. Methods

We collected data from the following trials: C-WORTHY (NCT01717326) [12, 13], C-SALVAGE (NCT02105454), [14] and C-EDGE (NCT02105467) [15]. We included patients infected with HCV with or without cirrhosis that received a fixed dose of 12 weeks or 18 weeks of GZR (100 mg) and EBR (50 mg), orally once-daily, with or without ribavirin for efficacy and safety analysis. Daily doses of ribavirin were based on the body weight of patients (51–65 kg, 800 mg/day; 66–80 kg, 1000 mg/day; 81–105 kg, 1200 mg/day; and >105 kg to 125 kg, 1400 mg/day), orally twice-daily in the morning and in the evening. Sustained virological response at 12 weeks (SVR12) after treatment and its two-sided 95% confidence intervals (CIs) were estimated. Comparisons between contingency tables were made by Fisher's exact test or chi-square test, with two-sided *P* value < 0.05 as significant.

## 3. Results

### 3.1. Baseline Characteristics

Data was pooled from three clinical trials conducted in the United States, Austria, Israel, Spain, Australia, Czech Republic, France, Germany, South Korea, Sweden, and Taiwan. There were four articles. Three were phase II and one was phase III.  Table 1 shows demographic characteristics of the patients included in this analysis. A total of 777 patients were enrolled in this analysis. Most of the patients were treatment-naive (73%), male (55%), white (80%), and with a median age of 56 years. Of these patients, 273 (35%) patients had cirrhosis. 432 (56%) patients had genotype 1a infection and 591 (76%) patients were* IL28B* non-CC.

These patients were divided into four groups by treatment regimen as follows: 422 (54%) received 12 weeks of grazoprevir plus elbasvir, 227 (29%) received grazoprevir plus elbasvir plus RBV, 63 (8%) received 18 weeks of grazoprevir plus elbasvir, and 65 (8%) received 18 weeks of grazoprevir plus elbasvir plus RBV.

### 3.2. Efficacy

95% (95% CI: 93–96, Table 2) of 777 patients achieved SVR12. The SVR12 rates for treatment-naive patients were 95% (95% CI: 92–96) and 96% (95% CI: 92–98) for previously treated patients. The SVR12 rates were 94% (95% CI: 92–96) for patients receiving 12 weeks of treatment and 97% (95% CI: 92–99) for those receiving 18 weeks of treatment. The rates were generally similar in subgroups, even with the cofactors of treatment duration and the regimen with or without RBV (Table 2), but SVR12 of the 33 treatment-experienced patients receiving 12 weeks of grazoprevir plus elbasvir without RBV was 91% (95% CI: 76–98). This was relatively lower than the 96%–100% SVR12 rates when the regimen added RBV or when the duration was extended to 18 weeks.

We merged three of the four included articles with related information for subgroup analysis (Table 3) [13–15]. And in the subgroup of age, only two articles were included [14, 15]. This analysis did not identify significant differences for comparisons of treatment-naive and previously treated patients for many factors including sex, age, race, HCV subtype,* IL28B* genotype, baseline HCV RNA, baseline NS3/4A, or NS5A RAS (*P* > 0.05). Two patients missing the data for* IL28B* genotype were excluded from this subgroup. NS3/4A RAS were identified at baseline in patients with genotype 1a or 1b infection; SVR12 was achieved in 138 of 145 and 206 of 213 patients with or without baseline RAS, respectively. Response was generally similar with or without NS3/4A RAS. NS5A RAS were also detected at baseline in genotype 1a or 1b infected patients. However, the rate of SVR12 was 99% (315/31, 95% CI: 97–100) in patients without baseline NS5A RAS, compared with 76% (34/45, 95% CI: 60–87) in patients with baseline NS5A RAS, without overlapping 95% CIs (*P* < 0.001) (Table 3).

### 3.3. Safety

Grazoprevir plus elbasvir was generally well-tolerated. Patients receiving 12 weeks and 18 weeks of grazoprevir plus elbasvir with RBV had more adverse events (AEs) than those receiving 12 weeks and 18 weeks of grazoprevir plus elbasvir alone. The most common AEs were fatigue, headache, nausea, and asthenia (Table 4). These events were more common in patients receiving RBV (29%, 20%, 14%, and 12%) than in those receiving grazoprevir plus elbasvir alone (18%, 20%, 8%, and 4%, resp.). And patients treated for 18 weeks had relatively higher rates of fatigue, headache, asthenia, nausea, and diarrhoea than those treated only for 12 weeks. In patients receiving grazoprevir plus elbasvir alone, the adverse events of headache and asthenia in patients treated for 18 weeks were significantly higher than those treated for 12 weeks (*P* = 0.015, *P* < 0.001, resp.). Besides, the rate of suffering at least one adverse event was significantly higher in 18-week treatment than 12-week when including RBV (*P* = 0.018).

Patients receiving grazoprevir plus elbasvir with or without RBV both had serious adverse events (SAEs) of 3% (13/484, 8/293, resp.). Two (<1%) SAEs were considered treatment-related according to the investigator (one case of abdominal pain, the other case of nausea). Overall, 3 (<1%) patients discontinued grazoprevir plus elbasvir and 4 (1%) discontinued grazoprevir plus elbasvir plus RBV because of an AE. Two patients receiving 12 weeks of grazoprevir plus elbasvir alone discontinued due to elevated liver aminotransferase levels and 1 discontinued because of palpitations and anxiety on treatment day 4. Three patients receiving 12 weeks of grazoprevir plus elbasvir plus RBV discontinued due to atrial fibrillation, drug intolerance, and death. One patient receiving 18 weeks of grazoprevir plus elbasvir plus RBV discontinued due to uterine bleeding.

## 4. Discussion

Most of the individuals (96%, 744/777) in this post hoc analysis are infected with HCV genotype 1. There were no differences between all subgroups for the rate of SVR12. Subgroup analysis showed no significance of sex, age, race, HCV subtype,* IL28B* genotype, or baseline HCV RNA on treatment outcome. High rates of SVR12 were shown across all groups regardless of adding RBV or extending treatment duration from 12 to 18 weeks. Specifically, the efficacy of 12 weeks of grazoprevir plus elbasvir without ribavirin was 95% (95% CI: 92–97) in treatment-naive patients and 91% (95% CI: 76–98) in treatment-experienced patients. Nevertheless, due to the difficulty of information extraction, few genotype 4 and 6 infected patients were also included in this analysis. The efficacy of these patients was lack of evidence at present; more patients need to be included in clinical trials to obtain sufficient evidence.

The treatment success in hard-to-cure patients can be used to evaluate the efficacy of a therapy. Treatment-naive patients with cirrhosis and previous null response patients with or without cirrhosis with genotype 1 infection were included in the C-WORTHY study. All patients receiving grazoprevir plus elbasvir with or without RBV achieved high rates of efficacy. The sustained virological response of 12 weeks of grazoprevir plus elbasvir without RBV was 97% in treatment-naive patients with cirrhosis, 91% in previous null response patients with or without RBV, and 92% in previous null response patients with cirrhosis [12]. For many therapies, sustained virological response can substantially decrease in treatment-experienced (peginterferon plus ribavirin), cirrhotic patients compared to those treatment-naive, noncirrhotic patients with high rate of SVR [6, 7, 16]. Among other all-oral regimens, there still remain some limitations. In a clinical trial of sofosbuvir plus ledipasvir with or without ribavirin, the overall efficacy in previously treated patients was 94–99%. However, the efficacy of 12-week ribavirin-free regimen was 86% in patients with cirrhosis and 87% in genotype 1b patients [17]. In patients receiving ABT-450 plus ritonavir plus ombitasvir plus dasabuvir plus ribavirin with cirrhosis, the SVR12 rates of 12 or 24 weeks of treatment were 92% or 96%, respectively, while adverse events increased [16]. In addition, a regimen of 12 weeks of simeprevir plus sofosbuvir with or without ribavirin in well compensated patients with cirrhosis showed the SVR12 rate of 89% [18]. The C-WORTHY study showed the high efficacy of NS3/4A protease inhibitor (grazoprevir) plus NS5A inhibitor (elbasvir), including the patients with poor response to other therapies [12]. However, most of the patients in this analysis were well compensated; the efficacy and safety of grazoprevir plus elbasvir in those with decompensated disease were not involved. A further study is urgent for such patients to evaluate the efficacy and safety of this regimen.

The prevalence of HCV infection is about one-third among HIV-infected patients [19]. 59 previously untreated coinfected individuals with HCV genotype 1 were also enrolled in the C-WORTHY trial. The response in monoinfected patients receiving 12 weeks of treatment was 93% with ribavirin and 98% without ribavirin, yet, in coinfected patients, the efficacy was 97% (28/29, 95% CI: 82–100) with ribavirin and 87% (87%, 95% CI: 69–96) without ribavirin [13]. There is no difference between monoinfected and coinfected patients or regimens with or without ribavirin based on the 95% CIs. Historical cross-study comparisons have demonstrated that HIV/HCV coinfection is one of the reasons for poor response to interferon-based HCV therapy. In large studies of peginterferon alfa-2a (APRICOT [20]) and alfa-2b (RIBAVIC [21]) plus ribavirin, response in coinfected patients with HCV genotype 1 was about half of that in monoinfected patients (29%, 20%, resp.) [22, 23]. The results of the C-WORTHY study suggest that grazoprevir plus elbasvir may be effective for both monoinfected and coinfected patients. The relatively small number of coinfected patients may be one of the limitations of the trial and the fairly easy-to-cure patients may be another.

According to this analysis, the regimen of grazoprevir plus elbasvir with or without ribavirin was well-tolerated. The frequency of serious adverse events and discontinuity because of adverse events was low (3% and <1%, resp.). The frequency was similar in patients with or without ribavirin. However, the incidence of adverse events was lower in ribavirin-free groups. Similarly, ribavirin also contributed to the adverse events [24]. Due to the high rates of SVR12 without ribavirin, the ideal regimen could be ribavirin-free.

The NS3/4A resistance-associated variants (RAS) at baseline did not affect the efficacy of grazoprevir plus elbasvir with or without ribavirin significantly. 95% (95% CI: 90–98) of the patients with NS3/4A RAS achieved SVR12. The association between baseline NS5A RAS and SVR12 was discovered. 99% (95% CI: 97–100) of the patients without baseline NS5A RAS achieved SVR12 while only 76% (95% CI: 60–87) of the patients with baseline NS5A RAS achieved SVR12. Therefore, preexisting of NS5A RAS may influence the efficacy obviously. Of course, this result was not representative due to lack of information.

The lack of innovation is a limitation of our study. However, it is still necessary to conduct this comprehensive analysis since grazoprevir plus elbasvir with or without ribavirin is a new all-oral therapy for HCV infection and it has not been approved in China. Second, this study increased the sample size; the results of this analysis were more representative and persuasive. In addition, HCV-1 genotype is the most common one and we only carried out this comprehensive analysis on it. The confounding factors of other genotypes were controlled effectively.

In summary, this analysis suggests that the oral fixed-dose combination of an NS3/4A protease inhibitor (grazoprevir) and NS5A inhibitor (elbasvir) is effective and well-tolerated for treatment-naive and previously treated patients with chronic genotype 1 HCV infection. Adding RBV or extending treatment duration may be of little benefit, except for treatment-experienced patients. The regimen of all-oral, 2-drug combination with or without RBV provides a new therapeutic option for chronic HCV infection.

## 5. Conclusion

12-week grazoprevir plus elbasvir therapy is safe and effective for treatment-naive patients with HCV genotype 1.

## Figures and Tables

**Table 1:** Baseline demographic characteristics.

Characteristic	Treatment-naive (*n* = 568)	Previously treated (*n* = 209)	Total (*n* = 777)
Mean age, years	56.5	54.3	55.9
Mean BMI, kg/m^2^	26.44	26.98	26.59
Sex			
Male, *n* (%)	308 (54.2)	120 (57.4)	428 (55.1)
Female, *n* (%)	260 (45.8)	89 (42.6)	349 (44.9)
Race			
White, *n* (%)	420 (73.9)	198 (94.7)	618 (79.5)
Nonwhite (*n*%)	148 (26.1)	11 (5.3)	159 (20.5)
Fibrosis stage			
Metavir F0–F2	330 (58.1)	100 (47.8)	430 (55.3)
Metavir F3	46 (8.1)	27 (12.9)	73 (9.4)
Metavir F4	192 (33.8)	82 (39.2)	274 (35.3)
HCV genotype, *n* (%)			
1a	326 (57.4)	106 (50.7)	432 (55.6)
1b	209 (36.8)	103 (49.3)	312 (40.2)
1-other	33 (5.8)	0	33 (4.2)
Mean HCV RNA, log_10_ (IU/mL)	6.39	6.49	6.42
*IL28B* CC genotype, *n* (%)			
CC	172 (97.7)	4 (2.3)	176 (22.7)
Non-CC	393 (66.5)	198 (33.5)	591 (76.1)
Unknown	3 (30.0)	7 (70.0)	10 (1.3)
Regimen received			
GZR-EBR 12 weeks	389 (68.5)	33 (15.8)	422 (54.3)
GZR-EBR + RBV 12 weeks	116 (20.4)	111 (53.1)	227 (29.2)
GZR-EBR 18 weeks	31 (5.5)	32 (15.3)	63 (8.1)
GZR-EBR + RBV 18 weeks	32 (5.6)	33 (15.8)	65 (8.4)

**Table 2:** SVR12 by baseline factors, treatment history, and regimen.

Response	Total (*n*/*N*, %)	Treatment-naive (*n*/*N*, %)	Previously treated (*n*/*N*, %)	*P* ^*∗*^
Overall (%)	737/777 (95)	537/568 (95)	200/209 (96)	0.519
95% CI	(93, 96)	(92, 96)	(92, 98)	
By treatment duration (%)				
12 weeks	613/649 (94)	477/505 (94)	136/144 (94)	0.996
18 weeks	124/128 (97)	60/63 (95)	64/65 (98)	0.361
By regimen (%)				
Without RBV	460/485 (95)	399/420 (95)	61/65 (94)	0.761
With RBV	277/292 (95)	138/148 (93)	139/144 (97)	0.204
By treatment duration + regimen (%)				
GZR-EBR 12 weeks	400/422 (95)	370/389 (95)	30/33 (91)	0.401
GZR-EBR + RBV 12 weeks	213/227 (94)	107/116 (92)	106/111 (96)	0.308
GZR-EBR 18 weeks	60/63 (95)	29/31 (94)	31/32 (97)	0.613
GZR-EBR + RBV 18 weeks	64/65 (99)	31/32 (97)	33/33 (100)	0.492

^*∗*^They were compared between treatment-naïve and previously treated groups.

**Table 3:** Subgroup analysis of SVR12.

Variables	Total, *n*/*N*	SVR12 (95% CI), %	Treatment-naive (*n*/*N*, %)	Previously treated (*n*/*N*, %)	*P* ^d^
Sex^*∗*^					
Male	260/280	93 (89–96)	217/234 (93)	43/46 (93)	1.000
Female	237/244	97 (94–99)	204/211 (97)	33/33 (100)	0.598
Age^a^					
≥65 years	39/40	98 (87–100)	29/29 (100)	10/11 (91)	0.275
<65 years	336/355	95 (92–97)	270/287 (95)	66/68 (97)	0.548
Race^*∗*^					
White	365/385	95 (92–97)	291/308 (94)	74/77 (96)	0.776
Nonwhite	132/139	95 (90–98)	130/137 (95)	2/2 (100)	1.000
Genotype^*∗*^					
1a	250/269	93 (89–96)	222/239 (93)	28/30 (93)	1.000
1b	219/225	97 (94–99)	171/176 (97)	48/49 (98)	1.000
1-other	28/30	93 (78–99)	28/30 (93)	0	—
*IL 28B* genotype^b^					
CC	127/136	93 (88–97)	125/134 (93)	2/2 (100)	1.000
Non-CC	368/385	96 (93–97)	294/308 (95)	74/77 (96)	1.000
Unknown	1/1	100 (25–100)	1/1 (100)	0	—
Baseline HCV RNA^*∗*^					
≤800000 IU/mL	153/156	98 (94–100)	126/127 (99)	27/29 (93)	0.089
>800000 IU/mL	344/368	93 (90–96)	295/318 (93)	49/50 (98)	0.225
NS3/4A RAS at baseline^c^					
Baseline RAS	138/145	95 (90–98)	107/111 (96)	31/34 (91)	0.355
No baseline RAS	206/213	97 (93–99)	162/169 (96)	44/44 (100)	0.349
NS5A RAS at baseline^c^					
Baseline RAS	34/45	76 (60–87)	28/37 (76)	6/8 (75)	1.000
No baseline RAS	315/318	99 (97–100)	245/247 (99)	70/71 (99)	0.533

^*∗*^Three articles reporting the variables were included [13–15].

^a^One of the three articles not reporting the variable was excluded [13].

^b^Three articles were included. Two patients were missing data for *IL28B* genotype and were excluded. SVR12 was achieved in 1 of these 2 patients (50.0%, CI: 1.3% to 98.7%).

^c^One of the three articles not reporting the variable was excluded [2], and only genotypes 1a and 1b were reported. One of 79 patients in study 4 was sequenced of NS5A but not sequenced of NS3/4A.

^d^They were compared between treatment-naïve and previously treated groups.

**Table 4:** Discontinuations and AEs by treatment regimen.

Variables	GZR-EBR				GZR-EBR + RBV			
	Total (*n* = 484)	12 weeks (*n* = 421)	18 weeks (*n* = 63)	*P* ^a^	Total (*n* = 293)	12 weeks (*n* = 228)	18 weeks (*n* = 65)	*P* ^b^
Discontinuation because of AE	3 (<1)	3 (<1)	0	1.000	4(1)	3 (1)	1 (2)	1.000
Any SAE	13 (3)	12 (3)	1 (2)	1.000	8(3)	7 (3)	1 (2)	0.690
Treatment-related SAE	1 (<1)	0	1 (2)	0.130	1(<1)	1 (<1)	0	1.000
Common AEs^*∗*^								
At least one adverse event	344 (71)	293 (70)	51 (81)	0.064	236 (81)	177 (78)	59 (91)	0.018
Fatigue	86 (18)	73 (17)	13 (21)	0.523	84 (29)	60 (26)	24 (37)	0.095
Headache	98 (20)	78 (19)	20 (32)	0.015	60 (20)	43 (19)	17 (26)	0.199
Nausea	41 (8)	37 (9)	4 (6)	0.517	41 (14)	32 (14)	9 (14)	0.969
Asthenia	18 (4)	9 (2)	9 (14)	<0.001	35 (12)	24 (11)	11 (17)	0.161

^*∗*^AEs: adverse events.

^a^They were compared in GZR-EBR treated patients between 12 weeks and 18 weeks.

^b^They were compared in GZR-EBR + RBV treated patients between 12 weeks and 18 weeks.
